# The Proliferation Index of Specific Bone Marrow Cell Compartments from Myelodysplastic Syndromes Is Associated with the Diagnostic and Patient Outcome

**DOI:** 10.1371/journal.pone.0044321

**Published:** 2012-08-31

**Authors:** Sergio Matarraz, Cristina Teodosio, Carlos Fernandez, Manuel Albors, María Jara-Acevedo, Antonio López, María Gonzalez-Gonzalez, María Laura Gutierrez, Juan Flores-Montero, Carlos Cerveró, Marlies Pizarro-Perea, María Paz Garrastazul, Gonzalo Caballero, Oliver Gutierrez, Guy Daniel Mendez, Manuel González-Silva, Paula Laranjeira, Alberto Orfao

**Affiliations:** 1 Centro de Investigación del Cáncer (Instituto de Biología Celular y Molecular del Cáncer, CSIC-USAL), IBSAL, Servicio de Citometría and Departamento de Medicina, Universidad de Salamanca, Salamanca, Spain; 2 Servicio de Hematología, Hospital Juan Canalejo, La Coruña, Spain; 3 Servicio de Hematología, Hospital Virgen de la Luz, Cuenca, Spain; 4 Servicio de Hematología, Hospital Lucus Augusti, Lugo, Spain; 5 Servicio de Hematología, Hospital Punta de Europa, Algeciras, Spain; 6 Servicio de Hematología, Hospital San Jorge, Huesca, Spain; 7 Servicio de Hematología, Hospital Rio Hortega, Valladolid, Spain; 8 Servicio de Hematología, Hospital de Jerez de la Frontera, Cádiz, Spain; 9 Servicio de Hematología, Hospital La Línea, Cádiz, Spain; University of Barcelona, Spain

## Abstract

Myelodysplastic syndromes (MDS) are clonal stem cell disorders which frequently show a hypercellular dysplastic bone marrow (BM) associated with inefficient hematopoiesis and peripheral cytopenias due to increased apoptosis and maturation blockades. Currently, little is known about the role of cell proliferation in compensating for the BM failure syndrome and in determining patient outcome. Here, we analyzed the proliferation index (PI) of different compartments of BM hematopoietic cells in 106 MDS patients compared to both normal/reactive BM (n = 94) and acute myeloid leukemia (AML; n = 30 cases) using multiparameter flow cytometry. Our results show abnormally increased overall BM proliferation profiles in MDS which significantly differ between early/low-risk and advanced/high-risk cases. Early/low-risk patients showed increased proliferation of non-lymphoid CD34^+^ precursors, maturing neutrophils and nucleated red blood cells (NRBC), while the PI of these compartments of BM precursors progressively fell below normal values towards AML levels in advanced/high-risk MDS. Decreased proliferation of non-lymphoid CD34^+^ and NRBC precursors was significantly associated with adverse disease features, shorter overall survival (OS) and transformation to AML, both in the whole series and when low- and high-risk MDS patients were separately considered, the PI of NRBC emerging as the most powerful independent predictor for OS and progression to AML. In conclusion, assessment of the PI of NRBC, and potentially also of other compartments of BM precursors (e.g.: myeloid CD34^+^ HPC), could significantly contribute to a better management of MDS.

## Introduction

Myelodysplastic syndromes (MDS) are heterogeneous clonal stem cell disorders characterized by dysplastic hematopoiesis leading to bone marrow (BM) failure and an increased risk of transformation into acute myeloid leukemia (AML). Typically, the disease is associated with impaired maturation and defective production of myeloid cells, which translates into dysplastic features, cytopenias and a remarkable negative impact on patient survival [1]. Current prognostic stratification of MDS is mainly based on the percentage of BM blast cells, the number of cytopenias and cytogenetics [2], together with hemoglobin levels and/or other more dynamic variables (e.g.: transfusion dependency) [1], [3]. However, currently used prognostic models based on these variables remain relatively limited, particularly for predicting the outcome of low risk MDS. Consequently, the search for additional prognostic factors allowing for more precise prognostic stratification and treatment selection of these patients remains a challenge. Other parameters such as a poor performance status together with an older age, leukocytosis, increased LDH serum levels and the number and severity of comorbidities [4], [5] have also been associated with a poor outcome in low-risk MDS, but their contribution to the prognostic models proposed so far still shows important limitations, as discussed elsewhere [6], [7].

The proliferation index (PI) of specific compartments of BM cells is a dynamic parameter that reflects the ongoing rate of production of hematopoietic cells in MDS, which can be easily assessed at any time during the course of the disease [8]. In addition, the PI is also directly related to the maturation-associated alterations of distinct subtypes of hematopoietic cells in individual patients [8]. In this regard, early studies already showed epigenetic repression of specific genes involved in the cell cycle and decreased numbers of S-phase cells in association with BM failure among advanced MDS and AML patients [9], [10], [11], [12], [13], [14], suggesting that assessment of the PI of BM cells in MDS may be of potential relevance for prognostic stratification and monitoring of the disease [15]. Despite this, information currently available about the PI of BM cells in MDS remains very limited and controversial, preliminary data in the literature suggesting that disease progression could be associated with both proliferation arrest and enhanced expansion of clonal cells [9], [14], [16], [17], [18]. However, careful analysis of these studies shows that many of them have focused on the assessment of the proliferation rate of the overall BM cellularity, which largely depends on the relative composition of the sample in distinct cell compartments; moreover, these studies are restricted to the analysis of a few BM cell compartments in relatively small and unstratified cohorts of MDS patients, without investigating its potential impact on the outcome of the disease [9], [11], [19].

**Table 1:** Proliferation index (percentage of S+G_2_M cells) of different BM cell compartments in MDS *vs*. both AML and normal/reactive BM.

*BM cell compartment*	Proliferation index of BM cell populations					
	Normal/reactive BM (N = 94)	*P-value* †	MDS (N = 106)	*P-value* ¥	AML (N = 30)	*P-value* *
**Whole BM**	7% (1–17.5%)	***<0.001***	9% (2–27%)	***0.008***	7% (0.3–17%)	***NS***
**Non-lymphoid CD34^+^ precursors**	15% (6–28%)	***0.05***	13% (0.6–36%)	***<0.001***	6% (0.07–19%)	***<0.001***
**Maturing neutrophils**	5% (0.5–12%)	***<0.001***	7% (2–26%)	***NS***	7% (0.3–17%)	***0.02***
** ** ***CD13^hi^/CD11b*** *^−^* ***cells***	21% (4–37%)	***0.01***	19% (6–51%)	***NS***	17.5% (2–54%)	***NS***
** ** ***CD13^lo/int^/CD11b*** *^−^* ***cells***	21% (12–36%)	***NS***	21% (5–46%)	***NS***	17% (3–41%)	***NS***
** ** ***CD13^lo/int^/CD11b^+^ cells***	4 (0.8–12%)	***0.002***	5% (1–23%)	***NS***	7% (1–43%)	***0.001***
** ** ***CD13^hi^/CD11b^+^ cells***	0.5% (0.1–4%)	***<0.001***	2% (0.3–12%)	***0.007***	2% (0.2–36%)	***<0.001***
**Monocytic cells**	4.5% (0.1–21%)	***NS***	4% (0–16%)	***NS***	7% (0–10%)	***NS***
**Nucleated red blood cell precursors**	28% (11–45%)	***NS***	26% (2–48%)	***0.01***	21% (2–37%)	***<0.001***
**Eosinophils**	4% (0–13%)	***0.01***	7% (0–21%)	***NS***	5.5% (1.5–16%)	***NS***

Results expressed as median percentage of S+G_2_/M cells and range between brackets. AML, acute myeloid leukemia; MDS, myelodysplastic syndrome; BM, bone marrow; NS, statistically not significantly different;

† , MDS *vs*. normal/reactive BM;

¥ , MDS *vs*. AML;

* , AML *vs.* normal/reactive BM.

In this study, we analyzed for the first time the cell cycle distribution of different compartments of BM hematopoietic cells –e.g.: CD34^+^ hematopoietic progenitor and precursor cells, maturing neutrophils and monocytic cells, mature lymphocytes, eosinophils and nucleated red blood cell precursors (NRBC)- in a relatively large cohort of 230 BM samples including 106 MDS patients, 30 AML and 94 normal/reactive BM samples. Overall, our results show altered BM proliferation profiles in MDS, which significantly differ in early/low-risk *vs.* advanced/high-risk subtypes of the disease: increased proliferation of myeloid CD34^+^ precursors, maturing neutrophils and NRBC in early phases of the disease, and progressively decreased PI in advanced MDS and AML. Noteworthy, a higher PI of non-lymphoid CD34^+^ cells and NRBC were both associated with a significantly longer overall survival (OS) and decreased risk of AML transformation, even among patients within the low and intermediate-1 IPSS risk categories, the PI of NRBC emerging as the most powerful prognostic factor for both OS and progression-free survival in MDS, independently of the haemoglobin levels and other classical prognostic variables.

**Table 2:** Proliferation index (percentage of S+G_2_M cells) of different compartments of bone marrow (BM) cells in MDS patients grouped according to the World Health Organization (WHO) classification and the International Prognostic Scoring System (IPSS).

*BM cell compartment*	Proliferation index of BM cell populations								
	WHO Subtype (n = 106)								
	RA (N = 10)	RCMD (N = 38)		RAEB-1 (N = 20)	RAEB-2 (N = 30)		MD/MPN (N = 8)		*P-value*
**Whole BM**	13%†† (6–27%)	9%†† (2–21%)		9.5%†† (3–22%)	8% (3–21%)		9.5%† (4–14%)		*0.01*
**Non-lymphoid CD34^+^ precursors**	21%†† (8–36)	17% (3–28%)		13.5% (2–30%)	7%†† (0.6–24%)		18% (4–26%)		*<0.001*
**Maturing neutrophils**	8%†† (6–26%)	6% (2–13%)		7%†† (2–18%)	7%†† (2–17%)		9%†† (4–11%)		*0.001*
*** CD13^hi^/CD11b*** *^−^* ***cells***	21% (19–51%)	19% (7–35%)		18%† (9–35%)	15%†† (6–33%)		25% (15–30%)		*0.001*
*** CD13^lo/int^/CD11b*** *^−^* ***cells***	27%† (15–46%)	23% (5–35%)		21% (10–39%)	14.5%†† (6.5–30%)		22% (10–29%)		*0.003*
*** CD13^lo/int^/CD11b^+^ cells***	8%†† (3–12%)	5%†† (0.6–14%)		4% (2–14%)	3.5% (0.6–23%)		9%†† (3–11%)		*0.03*
*** CD13^hi^/CD11b^+^ cells***	1%†† (0.4–12%)	1%†† (0.3–7%)		2%†† (0.3–12%)	2.5%†† (0.3–7%)		2%†† (1–3%)		*<0.001*
**Monocytic cells**	5% (1.4–9%)	5.5% (0–16%)		1%†† (0–11%)	2.7%† (0–6%)		6% (2–13%)		*0.05*
**Nucleated red blood cell precursors**	31% (24–48%)	28.5% (2–44%)		27% (12–42%)	18%†† (4.5–42%)		29% (16–40%)		*0.001*
**Eosinophils**	3% (1–12%)	7% (0–21%)		5.5% (2–17%)	4% (0–14%)		4.5% (0–16%)		*NS*
***BM cell compartment***	**IPSS category (n = 85)**								
	**Low risk (N = 21)**		**INT-1 (N = 35)**	**INT-2 (N = 15)**		**High risk (N = 14)**		***P-value***	
**Whole BM**	9%†† (5–27%)		8% (2–22.5%)	11%†† (3.5–21%)		8% (3–12.5%)		*0.007*	
**Non-lymphoid CD34^+^ precursors**	22.5%†† (4–36%)		13% (2–30%)	12%†† (0.6–24%)		7%†† (1–11%)		*<0.001*	
**Maturing neutrophils**	8%†† (3–26%)		6% (2–14%)	6.5%† (2.5–18%)		7%†† (3.5–12%)		*0.005*	
*** CD13^hi^/CD11b*** *^−^* ***cells***	25% (7–51%)		18%†† (6–35%)	18%†† (6–29%)		13%†† (8–25%)		*0.001*	
*** CD13^lo/int^/CD11b*** *^−^* ***cells***	25%†† (10–46%)		21% (5–39%)	17%†† (7–27%)		15% (6.5–30%)		*0.005*	
*** CD13^lo/int^/CD11b^+^ cells***	7%† (2–12%)		5%†† (0.6–14%)	3.5% (1.5–15.5%)		4% (2–10%)		*NS*	
*** CD13^hi^/CD11b^+^ cells***	1.5%†† (0.3–12%)		1%†† (0.3–7%)	2%†† (0.3–12%)		3%†† (0.3–7.5%)		*<0.001*	
**Monocytic cells**	5% (0–13%)		2%†† (0–11%)	6% (1–16%)		3.5% (0–5%)		*NS*	
**Nucleated red blood cell precursors**	29% (16–48%)		28% (2–44%)	27%† (4.5–39%)		18%†† (7–42%)		*0.007*	
**Eosinophils**	4.5% (1–12%)		6% (0–21%)	6% (0–14%)		4% (0–10%)		*NS*	

Results expressed as median percentage of S+G_2_ M cells and range between brackets. RA, refractory anemia; RCMD, refractory cytopenia with multilineage dysplasia; RAEB, RA with excess of blasts; MD/MPN, myelodysplastic/myeloproliferative neoplasms; INT, intermediate risk; NS, statistically not significantly different.

† , p<0.05 and

†† , p<0.03 *vs.* normal/reactive BM.

## Materials and Methods

### Bone Marrow Samples

A total of 136 untreated patients (78 men and 58 women; mean age of 73 years, ranging from 38 to 87 years) with newly diagnosed MDS (n = 106) and AML not otherwise specified (NOS; n = 30), were included in the present study. According to the World Health Organization (WHO) criteria [20], MDS patients were classified as follows: RA, 7 patients; RA with ringed sideroblasts (-RS), 3; RCMD, 31; RCMD-RS, 7; RAEB-1, 20; RAEB-2, 30; myelodysplastic/myeloproliferative neoplasms (MD/MPN), 8 cases. According to the International Prognostic Scoring System (IPSS) [2], 25% of cases were classified as low-risk MDS, 41% as intermediate-1, 18% as intermediate-2 and 16% as high-risk MDS. Distribution of AML NOS cases, according to the WHO 2008 was as follows: AML with minimal differentiation, 3 patients; AML without maturation, 4; AML with maturation, 8; AML with myelodysplasia-related changes, 6; AML with myelomonocytic/monocytic maturation, 5. The other 4 cases were classified as mixed phenotype AL (T/myeloid, NOS). For multiparameter flow cytometry immunophenotypic studies, EDTA-anticoagulated BM samples were obtained at diagnosis from all MDS and AML cases. Moreover, an additional validation cohort of 11 MDS patients was analyzed both at diagnosis and at the last follow-up BM (mean follow-up time of 11±9 months; range: 4 to 36 months). Five of these 11 patients (RCMD, 3; RAEB-1, 1; RAEB-2, 1) had the same WHO diagnostic subtype at diagnosis and follow-up, while 4 (RCMD, 2; RAEB-1, 1; RAEB-2, 1) presented with more advanced disease than at diagnosis (AML, RAEB-1, AML and AML respectively). The remaining two patients (RCMD, 1; RAEB-1, 1) were studied both at diagnosis and after treatment when they had achieved morphological and cytogenetic remission at the time of follow-up.

In parallel, 94 freshly obtained EDTA-anticoagulated normal (n = 47) and reactive (n = 47) BM samples from an identical number of individuals (46 men and 48 women; mean age of 67 years; range, 47 to 79 years) were collected at the University Hospital of Salamanca (Spain). Normal BM samples were obtained from healthy donors and individuals undergoing orthopedic surgery, while reactive samples corresponded to patients with carential and megaloblastic anemias and other toxic (e.g.: drug-induced) or reactive cytopenias, including infection-associated leukopenias. None of the reactive samples showed clonal hematopoiesis, based on the absence of cytogenetic abnormalities –e.g., trisomy 8, −7/7q^−^, −5/5q^−^, del(20q) or −Y- as assessed by fluorescence in situ hybridization (FISH) and/or a polyclonal pattern of inactivation of chromosome X in females, evaluated by the human androgen receptor assay (HUMARA) in FACS-purified (≥97% purity) maturing neutrophils, monocytic cells, NRBC, CD34^+^ hematopoietic progenitor and precursor cells (HPC) and mature lymphocytes.

All BM samples were systematically studied within the first 18 hours after they were drawn, after written informed consent was given by each subject according to the recommendations of the local Ethics Committee -Comisión de Bioética of Centro de Investigación del Cáncer-IBMCC (USAL-CSIC)-, which approved the study, and to the principles expressed in the Declaration of Helsinki.

**Table 3:** Proliferation index (percentage of S+G_2_M cells) of different BM cell compartments in normal/reactive BM (n = 94) and MDS patients (n = 106) with normal/favourable (n = 83) versus intermediate/poor (n = 23) cytogenetics.

*Bone marrow cell subsets*		MDS	
	*Normal/reactive bone marrow*	*Normal/favourable cytogenetics*	*Intermediate/poor cytogenetics*
	*(n = 94)*	*(n = 83)*	*(n = 23)*
**Whole BM**	7% (1–17.5%)	9%** (2–27%)	9%** (3–22%)
**Non-lymphoid CD34^+^ precursors**	15% (6–28%)	14% (0.6–36%)	8%** ‡‡ (1–30%)
**Maturing neutrophils**	5% (0.5–12%)	7%** (2–26%)	6.5%** (3.5–18%)
* CD13^hi^/CD11b^−^ cells*	21% (4–37%)	19%* (6–51%)	18%** (7–50%)
* CD13^lo/int^/CD11b^−^ cells*	21% (12–36%)	22% (5–46%)	20% (6.5–41%)
* CD13^lo/int^/CD11b^+^ cells*	4% (0.8–12%)	5%** (0.6–16%)	4% (2–23%)
* CD13^hi^/CD11b^+^ cells*	0.5% (0.1–4%)	2%** (0.3–12%)	3%** ‡‡ (0.4–12%)
**Monocytic cells**	4.5% (0.1–21%)	3.5% (0–11%)	2.5% (0–16%)
**Nucleated red blood cell precursors**	28% (11–45%)	28% (2–48%)	22%** ‡‡ (4–39%)
**Eosinophils**	4% (0–13%)	6.5%** (0–21%)	5.5% (0–17%)
**Mature lymphocytes**	0.0±0.0%	0.0±0.0%	0.0±0.0%

Results expressed as median percentage of cells and range between brackets.

* , p<0.05 and

** , p<0.03 *vs.* normal/reactive BM; and;

‡‡ **,** p<0.03 *vs.* normal/favourable karyotype. Normal/favourable cytogenetics includes cases with a normal karyotype, -Y, del(5q) or isolated del(7q); poor cytogenetics includes cases with complex (≥3 chromosomal abnormalities) karyotypes and alterations of chromosome 7, except isolated del(7q), and; intermediate cytogenetics: other karyotypic abnormalities.

### Cell Cycle Analyses

Analysis of the distribution of different compartments of BM cells along the *G_0_/G1* and *S* plus *G_2_/M* cell cycle phases (PI) was performed immediately after samples were obtained using triple-stainings for nuclear DNA and two cell surface markers. Briefly, whole BM samples –cell concentration adjusted with phosphate buffered saline containing 0.5% bovine serum albumin (PBS/BSA; pH = 7.4) to 10^6^ cells in 100 µL/tube- were incubated in two separate aliquots for 10 min in the dark at room temperature (RT) with saturating amounts of the following combinations of fluorescein isothiocyanate (FITC)−/phycoerythrin (PE)-conjugated monoclonal antibodies (MAb) purchased from Becton Dickinson Biosciences (BD, San Jose, CA, USA): CD45/CD34 and CD11b/CD13. In order to control for unspecific binding of antibodies mature lymphocytes and neutrophils were used as an internal reference. [21] After lysing non-NRBC, nucleated cells were washed and resuspended in 0.5 mL PBS/BSA. Then, 3 µl of DRAQ5™ (Vitro SA, Madrid, Spain) was added to each tube, another incubation performed for 10 min in the dark (RT) and the sample aliquots were immediately run in a FACSCanto II™ flow cytometer (BDB) using the FACSDiva software program (BD). For each sample aliquot, information about >1×10^5^ cells corresponding to the whole BM cellularity was measured and stored. For data analysis, the INFINICYT™ software program (Cytognos SL, Salamanca, Spain) was used. The overall percentage of proliferating cells, including those cells within the S plus G_2_/M cell cycle phases (PI), was defined as those cells showing a brighter staining of DRAQ5 than those included in the G_0_/G_1_ peak, as described elsewhere [22], [23].

In every sample, the following cell populations were identified after excluding dead cells and cell doublets in a sideward light scatter (SSC) *versus* DRAQ5-fluorescence area and a DRAQ5-fluorescence area *versus* DRAQ5-fluorescence width bivariate dot plot, respectively: total CD34^+^ HPC (CD45^lo^ CD34^+^ events), CD34^+^ non-lymphoid (e.g.: myeloid) HPC (SSC^int^ CD34^+^), CD34^+^ lymphoid HPC (SSC^lo^ CD34^+^ cells), nucleated red cell precursors (SSC^lo/very−lo^ CD45^−^ events), monocytic cells (SSC^int^ CD45^int/hi^ CD11b^hi^ CD13^hi^ cells), eosinophils (SSC^hi^ autofluorescent cells), mature lymphocytes (CD45^hi^ SSC^lo^) and maturing neutrophils (CD45^lo^ SSC^int/hi^); in addition, maturing neutrophils were further subdivided into four maturation-associated cell subsets, as previously described: [24] CD13^hi^ CD11b^−^, CD13^lo/int^ CD11b^−^, CD13^lo/int^ CD11b^+^ and CD13^hi^ CD11b^+^ neutrophil precursors. The specific immunophenotypic characteristics of mature lymphocytes were taken as a standard to define the relative position of the different compartments of BM precursors in a CD45 versus SSC bivariate dot plot. For each of the above listed cell populations, the distribution along the *G_0_/G1* and *S* plus *G_2_/M* cell cycle phases (proliferation index) was calculated, as described above.

### Conventional Karyotyping, Fluorescence in situ Hybridization (FISH) and HUMARA Studies

Cytogenetic analysis of BM samples was performed according to standard procedures [25] and interpreted using the International System for Cytogenetic Nomenclature criteria [26]. In addition, multicolor interphase FISH (iFISH) [27] was systematically performed for the detection of the most frequent recurrent cytogenetic abnormalities in MDS, using the following chromosomal probes (all purchased from Vysis Inc, Downers Grove, IL, USA): 1) LSI D5S23, D5S71 Spectrum Green (SG)/LSI EGFR Spectrum Orange (SO) probe combination for chromosome 5; 2) LSI D7S486 (7q31) SO/CEP 7 SG probes for chromosome 7; 3) CEP 8 (D8Z2) SO/CEP Y (DYZ1) SG probes for chromosomes 8 and Y, respectively, and; 4) LSI D20S108 (20q12) SO probe for chromosome 20. Investigation of the pattern of inactivation of chromosome X was analyzed in FACS-purified (FACSAria, BDB) (purity≥97%) maturing neutrophils, monocytic cells, NRBC, CD34^+^ HPC and mature lymphocytes, as previously reported. [28].

**Table 4:** Relationship between the degree of proliferation of non-lymphoid CD34^+^ and nucleated red blood cells from patients with myelodysplastic syndromes (MDS) and other haematological and biochemical characteristics of the disease.

*MDS patient subgroups*	PI of non-lymphoid CD34^+^ precursors			PI of nucleated red blood cells		
	PI ≥10%	PI <10%	*P-value*	PI ≥24%	PI <24%	*P-value*
**N. of cytopenias (≥2)** *(n = 84)*	29/52 (56%)	27/32 (84%)	***0.007***	28/49 (57%)	28/35 (80%)	***0.02***
**Anemia (<100 g haemoglobin/L)** *(n = 83)*	28/52 (54%)	26/31 (84%)	***0.006***	26/49 (53%)	28/34 (82%)	***0.006***
**Thrombocytopenia (<100×10^9^ platelets/L)** *(n = 84)*	19/52 (36.5%)	22/32 (70%)	***0.004***	17/49 (35%)	24/35 (70%)	***0.002***
**Leukopenia (<5×10^9^ leukocytes/L)** *(n = 84)*	26/52 (50%)	18/32 (56%)	***NS***	26/49 (53%)	18/35 (51%)	***NS***
**Increased LDH (>400 IU/L)** *(n = 72)*	6/43 (14%)	13/29 (45%)	***0.004***	7/40 (18%)	12/32 (38%)	***0.05***
**Neutropenia (<1.8×10^9^ neutrophils/L)** *(n = 64)*	19/37 (52%)	18/27 (67%)	***NS***	17/35 (48.5%)	20/29 (70%)	***NS***
**Transfusion dependency** *(n = 65)*	16/40 (40%)	21/25 (84%)	***<0.001***	12/36 (33%)	25/29 (86%)	***<0.001***
**Intermediate/poor cytogenetics** *(n = 98)*	6/63 (10%)	16/35 (46%)	***<0.001***	9/60 (15%)	13/38 (34%)	***0.02***
**Evolution to AL** *(n = 77)*	6/48 (12%)	11/29 (38%)	***0.009***	5/46 (10%)	12/31 (40%)	***0.004***

Results expressed as number of cases from all MDS patients analyzed and percentage between brackets.

PI, proliferation index;

LDH, lactate dehydrogenase;

AL, acute leukemia;

NS, statistically not significantly different.

### Statistical Methods

For continuous variables, mean values and standard deviation (SD), median and range were calculated using the SPSS software (SPSS 10.0, Chicago, IL); for categorical variables, frequencies were used (SPSS software). Parametric *vs*. non-parametric data distribution was assessed by the Kolmogorov-Smirnov (K-S) test. For categorical and continuous variables, comparisons between two or more groups were made using the χ^2^, and either the Student T (for parametric data) or the Mann-Whitney U and the Kruskal-Wallis tests (for non-parametric data), respectively. Receiver operating characteristic (ROC) curves were applied for the definition of PI cut-off values for association with e.g. cytopenias and survival. Survival curves were plotted according to the method of Kaplan and Meier [29] and compared by the log-rank test. Based on those variables showing a significant effect on overall survival in the univariate analysis, a multivariate Cox proportional-hazards model was constructed with those parameters showing independent predictive value; inclusion in the final model was determined by a backward stepwise approach. *P* values <0.05 were considered to be associated with statistical significance.

**Figure 1:**
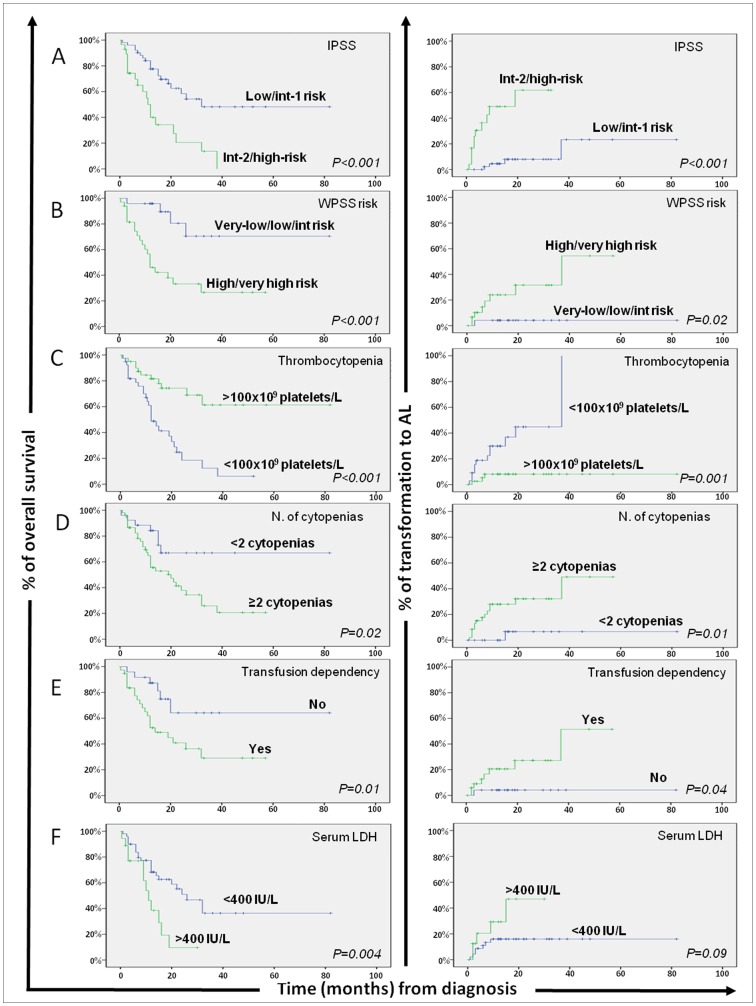
Impact of currently used prognostic classifications and other disease features on overall survival and risk of transformation to acute leukemia (AL) of patients with myelodysplastic syndromes (MDS; n = 106). Overall survival (left column) and progression-free survival (transformation to AL; right column) curves are plotted for patients with MDS grouped according to the International Prognostic Scoring System (IPSS; row A), the World Health Organization-based Scoring System (WPSS; row B), the number of peripheral blood platelets at diagnosis (row C), the presence of multiple cytopenias (row D), transfusion dependency (row E), and serum LDH levels (row F).

**Table 5:** Prognostic factors for overall survival (OS) and progression (transformation to acute leukemia)-free survival (PFS) in MDS.

*Prognostic factor*			Overall survival			Evolution to AL	
	Adverse category	Median OS* (range)	Univariate analysis	Multivariate analysis	Median PFS (range)	Univariate analysis	Multivariate analysis
			*P-value*	*P-value (HR)*		*P-value*	*P-value (HR)*
***Anemia***	<100 g haemoglobin/L	21 (13–29)	–	–	Not reached	–	–
***Thrombocytopenia***	<100×10^9^ platelets/L	12 (9–15.4)	<0.001	0.008 (2.7)	37 (6–68)	0.001	–
***≥2 Cytopenias***	Yes	20 (8–32)	0.02	–	37 (7–67)	0.01	–
***Serum LDH***	>400 IU/L	11 (7.5–14.5)	0.004	–	Not reached	–	–
***Transfusion dependency***	Yes	14 (6–22)	0.01	–	37 (11.5–62.5)	0.04	–
***IPSS***	Intermediate-2/high risk groups	11 (9–13)	<0.001	–	19 (3–35)	<0.001	
***WPSS***	High/very-high risk groups	12 (8–16)	<0.001		37 months	0.02	
***PI of non-lymphoid CD34^+^ precursors***	<10%	12 (7.5–16.4)	0.005	–	37 months	0.001	–
***PI of NRBC***	<24%	10.5 (6.4–14.5)	<0.001	0.005 (3.7)	19 (0–40)	<0.001	0.01 (12.3)

PI, proliferation index; NRBC, nucleated red blood cells; LDH, lactate dehydrogenase; AL, acute leukemia;

* OS, overall survival expressed in months; PFS: progression to acute leukemia-free survival expressed in months; HR, hazard ratio.

**Figure 2:**
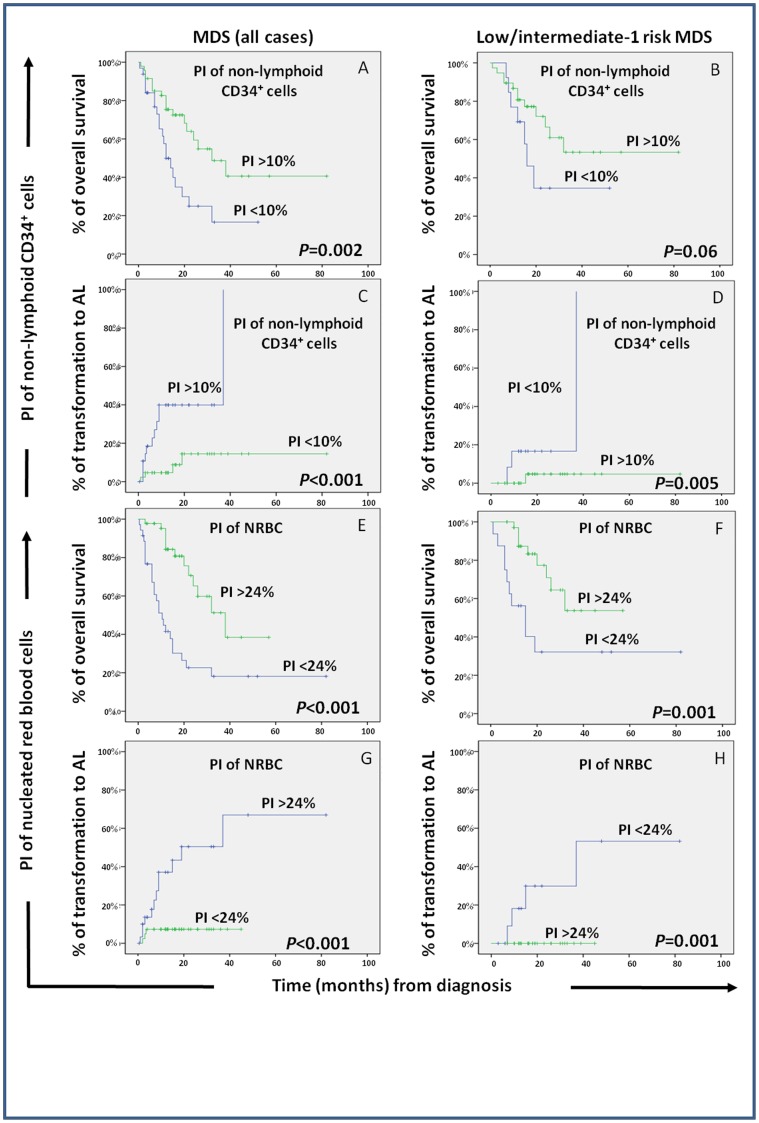
Impact of the proliferation index (PI) of BM non-lymphoid (e.g.: myeloid plus immature) CD34^+^ precursors and nucleated red blood cells (NRBC) on the overall survival and progression (transformation to acute leukemia; AL) free survival of patients with myelodysplastic syndromes (MDS). Overall survival and progression-free survival curves are plotted for groups of MDS patients classified according to the PI of non-lymphoid (e.g.: myeloid plus immature) CD34^+^ cells (panels A to D) and NRBC (panels E to H). In the left column all MDS patients (n = 106) are analyzed together, while in the right column only MDS patients in the low plus intermediate-1 IPSS risk categories (n = 56) are considered.

## Results

### Proliferation of Hematopoietic Cells in Normal/reactive BM

We have recently shown that similar PI are found in normal *vs.* reactive BM [8], as confirmed here (Table 1) for the overall BM cellularity and the distinct compartments of maturing hematopoietic cells, except for non-lymphoid (myeloid plus immature) CD34^+^ HPC (16±4% *vs.* 14±5%, p = 0.01) and monocytic cells (4.6±4.5% *vs*. 6±3.5%, p = 0.03). Therefore, from now on normal and reactive BM samples will be considered together (n = 94) for further evaluation of potential alterations in the PI of BM cells in MDS.

### Overall Proliferation of Hematopoietic Cells in MDS vs Normal/reactive and AML BM

Overall, significantly increased numbers of S+G_2_ M phase cells (PI) were detected in whole BM of MDS patients *vs.* controls (median PI of 9% *vs*. 7%, p<0.001). Such increased proliferation was due to increased PI of maturing neutrophils (p<0.001) at the expense of the more mature CD11b^+^ granulocytes (p≤0.002) (Table 1). By contrast, both non-lymphoid CD34^+^ HPC and the more immature CD13^hi^/CD11b^−^ neutrophil precursors showed significantly lower PI in MDS *vs.* normal/reactive BM (p≤0.05) (Table 1).

In turn, AML NOS cases showed an overall BM proliferation rate similar to normal/reactive BM, but significantly lower than that of MDS (PI of 7% *vs.* 9%, respectively; p = 0.008) (Table 1). In detail, AML showed a pronounced decrease of the PI of non-lymphoid CD34^+^ cells (p<0.001) and NRBC (p≤0.01) *vs*. both normal/reactive BM and MDS. In turn, a marked increased proliferation was detected in AML patients among the CD13^lo/int^/CD11b^+^ and CD13^hi^/CD11b^+^ subsets of maturing neutrophils *vs*. normal/reactive BM (p≤0.001), the latter subset also showing an increased PI *vs*. MDS (p = 0.007) (Table 1).

### Proliferation of BM Cells in Different Diagnostic Subtypes of MDS

Despite the increased overall BM proliferation detected in MDS *vs*. both AML and normal/reactive BM, distinct proliferation profiles were observed for the different BM cell compartments among the diagnostic and prognostic subgroups of MDS (Table 2), except for RA and RCMD patients with *vs*. without ring sideroblasts; therefore, these two WHO subtypes of MDS were grouped together.

Overall, the highest BM PI was observed among RA and low-risk MDS patients (13% and 9% *vs*. 7%, p≤0.006). Such increased proliferation was associated with a greater PI of non-lymphoid CD34^+^ precursors in both subgroups of early MDS patients (p≤0.006). However, the PI of these precursors showed a trend to decline in high-risk MDS, reaching minimal proliferation values in RAEB-2, intermediate-2, high-risk IPSS MDS and AML patients (p≤0.01 *vs*. normal BM, RA and low-risk MDS) (Table 2).

Similarly, despite a significantly enhanced proliferation of maturing BM neutrophils was found in all diagnostic categories of MDS but RCMD (p≤0.01), as well as in AML (p≤0.02), a more detailed analysis of the neutrophil maturation stages (Table 2), revealed again significantly different proliferation profiles in early *vs*. advanced MDS/AML, particularly among the more immature neutrophil precursors. Thus, all MDS diagnostic categories showed a significantly increased PI for the more mature CD13^hi^/CD11b^+^ and/or CD13^lo/int^/CD11b^+^ neutrophil subsets (p≤0.04); by contrast, although a similarly increased (p≤0.03) or stable PI was detected among immature CD11b^−^ neutrophil precursors from RA and low-risk IPSS MDS, decreased PI (*vs*. normal/reactive BM) were found for these cells (CD13^hi^/CD11b^−^ myeloblasts) in RAEB-1 and both intermediate-1- and high-risk IPSS MDS (p≤0.04) as well as (CD13^hi^/CD11b^−^ myeloblasts and CD13^lo/int^/CD11b^−^ promyelocytes) in RAEB-2 and intermediate-2 IPSS MDS cases (p≤0.02). Of note, BM cells from MD/MPN patients showed proliferation patterns similar to those of early/low-risk MDS cases, while in AML they resembled those of advanced/high-risk MDS patients (Table 2).

In addition, altered proliferation patterns were also observed among BM NRBC from both MDS and AML patients: the PI of NRBC tended to increase in RA patients (p>0.05 *vs.* normal/reactive BM) while it significantly declined in intermediate-2- (p = 0.05), RAEB-2 (p<0.001), high-risk MDS (p<0.001), and in AML (p<0.001) (Tables 1 and 2). In turn, the cell cycle distribution of monocytic cells remained at normal/reactive levels in early MDS and AML, while abnormally lower PI were observed in RAEB-1, intermediate-1-risk and RAEB-2 patients (p≤0.05 *vs.* normal/reactive BM); conversely, a tendency towards increased monocytic proliferation was detected in MD/MPN (p>0.05).

No significant differences were noted in the PI of other BM cell compartments analyzed (e.g.: B-cell committed CD34^+^ precursors) neither in MDS nor in AML; mature lymphocytes systematically corresponded to resting cells in all groups analyzed.

Although variable results were observed for individual cases, follow-up studies performed in a group of 11 MDS patients showed a tendency towards decreased PI of non-lymphoid CD34^+^ cells –from 5% (0–17%) to 0.9% (0–14%); p = 0.06-, CD11b^−^ neutrophil precursors –from 30% (8–38%) to 16% (4–28%); p = 0.02- and NRBC –from 28% (9–32%) to 19% (7–30%); p = 0.05- *vs*. those found at diagnosis; such tendency was observed not only among cases which showed more advanced disease but also among cases which had the same WHO diagnosis after follow-up (n = 5, data not shown). The only exception corresponded to those two cases who achieved remission, in which the PI of the different compartments of BM cells returned to normal BM values: PI of non-lymphoid CD34^+^ cells, CD11b^−^ neutrophil precursors and NRBC of 6% (3–9%), 11.5% (8–15%) and 19% (13–25%) vs. 15% (14–16%), 32.5% (22–43%) and 27% (26–28%), respectively.

### Cell Cycle Distribution of BM Cells in Different Cytogenetic Subgroups of MDS

MDS cases with normal/favourable karyotypes as well as cases with isolated del(7q) showed similar proliferation profiles consisting of an enhanced BM proliferation (p = 0.007) due to an increased PI of the more mature neutrophils (p = 0.01) in the absence of altered cell cycle profiles among non-lymphoid CD34^+^ and NRBC precursors (Table 3). Conversely, patients with intermediate/poor cytogenetics had decreased PI of non-lymphoid CD34^+^ and NRBC *vs*. both normal/reactive BM (p≤0.002) and MDS cases with normal/favourable cytogenetics (p = 0.01) (Table 3).

### Relationship between the PI of BM Cell Compartments in MDS and other Features of the Disease

Upon grouping MDS patients according to the PI of BM cells (Table 4), a clear association was observed between a lower PI of non-lymphoid CD34+ precursors (PI: <10% vs ≥10%) and other relevant features of the disease such as anemia (p = 0.006), thrombocytopenia (p = 0.004) and multiple (≥2) cytopenias (p = 0.007), transfusion dependency (p<0.001), increased serum LDH (p = 0.004), intermediate/poor cytogenetics (p<0.001) and transformation to acute leukemia (AL) (p = 0.009). Likewise, low NRBC PI (<24% vs ≥24%) were also associated with a greater frequency of ≥2 cytopenias (p = 0.02), anemia (p = 0.006), thrombocytopenia (p = 0.002), transfusion dependency (p<0.001), increased LDH (p = 0.05), intermediate/poor karyotypes (p = 0.02) and transformation to AL (p = 0.004) (Table 4). Interestingly, a low PI (<12.5% and <11.7%) of non-lymphoid CD34^+^ cells could predict with a great efficiency among MDS cases for the presence of anemia and thrombocytopenia, respectively (sensitivity of 63% and specificity of 80% in both cases; p≤0.02); likewise, a PI of NRBC <24.5% also predicted for anemia with a high specificity (sensitivity of 49%, specificity of 80%; p = 0.05), while a low PI of CD13^hi^/CD11b^−^ maturing neutrophils efficiently identified patients with neutropenia (sensitivity of 65%, specificity of 70%; p = 0.03).

### Impact of Cell Proliferation on the Outcome of MDS

From the prognostic point of view, a significantly shorter median overall survival (OS) was observed among MDS cases which displayed lower percentages of proliferating non-lymphoid CD34^+^ precursors (p = 0.005) and NRBC (p<0.001), thrombocytopenia (p<0.001), ≥2 cytopenias (p = 0.02), increased serum LDH (p = 0.004), transfusion dependency (p = 0.01) and higher IPSS and WPSS scores (p<0.001). In turn, a lower percentage of proliferating non-lymphoid CD34^+^ precursors (p = 0.001) and NRBC (p<0.001), together with the presence of thrombocytopenia (p = 0.001), ≥2 cytopenias (p = 0.01), transfusion dependency (p = 0.04) and higher IPSS or WPSS scores (p≤0.02), were also associated with a greater risk of transformation to AL (Figure 1 and Table 5). Most interestingly, low- and intermediate-1 risk MDS patients with abnormally lower PI of non-lymphoid CD34^+^ precursors and NRBC showed a significantly lower median OS -16 vs 132 (p = 0.03) and 15 vs 132 months (p = 0.001), respectively- and a higher risk of transformation to AL –median of 37 vs 135 (p = 0.005) and of 37 vs 135 months (p = 0.002), respectively- (Figure 2). Meanwhile, intermediate-2 and high-risk MDS with low PI of NRBC displayed significantly shorter survival rates (median OS of 9 vs 22 months, respectively; p = 0.03) (Figure 2). Multivariate analysis of prognostic factors (Table 5) showed that the combination of the PI of NRBC –hazard ratio (HR) of 3.7; 95% CI of 2–7; p = 0.005) and thrombocytopenia (HR of 2.7; 95% CI of 1.3–6; p = 0.008), were the only two parameters showing independent predictive value for OS in MDS, while the PI of NRBC was the most informative independent predictor for transformation of MDS to AL both as categorical (HR of 12.3; 95% CI of 1.5–99.5; p = 0.01), and as a continuous variable (p = 0.01).

## Discussion

Prediction of outcome in MDS by conventional prognostic stratification models remains only partially successful. This is mainly due to the heterogeneous clinical behaviour and response to treatment observed, particularly among low-risk cases. Because of this, an increasing interest exists on the identification of new prognostic factors that could provide already at diagnosis, a more dynamic assessment of the behaviour of the disease and contribute to refine the currently used classifications to improve the management of individual patients [6], [7]. Although several parameters related to BM failure (e.g.: hemoglobin levels, transfusion needs, number of cytopenias) are significantly associated with the prognosis of the disease, and evidence exists about the potential involvement of cell proliferation in the pathogenesis of MDS, to the best of our knowledge, no study has been reported so far in which the proliferation profile of different compartments of BM cells is analyzed in MDS, and correlated with disease outcome.

Here, we investigated for the first time the proliferation rate of different compartments of BM hematopoietic cells in MDS *vs.* both normal/reactive BM and AML. Overall, an increased proliferation of BM cells was found in MDS *vs*. the other two groups, in line with previous observations [30], [31]. Specific analysis of the PI of distinct BM cell compartments revealed that such increased proliferation was mainly due to a higher PI of CD11b^+^ maturing neutrophils which could be viewed as an attempt to compensate for the need to produce mature neutrophils, required to maintain homeostatic levels of these cells in peripheral blood (PB). However, more detailed analysis of the PI of these and other BM cell compartments within the distinct diagnostic and prognostic subtypes of MDS highlighted the existence of significantly different proliferation profiles in low- *vs*. high-risk cases. Accordingly, early/low-risk MDS patients (RA and low-risk IPSS MDS) typically showed an overall increased proliferation of BM cells at the expenses of CD34^+^ non-lymphoid (myeloid plus uncommitted) precursors, maturing neutrophils and NRBC; conversely, advanced/high-risk patients showed progressive collapse of proliferation of these cell compartments, except for the more mature CD11b^+^ neutrophil-lineage cells, similarly to what was found in AML. These observations are in line with the more severe and numerous cytopenias found among the latter group of MDS patients, as well as with the hypermethylated status of cell cycle controlling genes (e.g.: p15^INK4b^, *CDKN2B*) reported to be associated with an enhanced hematopoiesis and neutrophil differentiation among specific subtypes of low-risk MDS (e.g.: RA with ringed sideroblasts) [32], [33], [34]. These differences in the proliferation rate of early/low-risk *vs.* advanced/high-risk MDS patients may be also related to overexpression of cell cycle-associated genes in precursor cells from early MDS (e.g.: cyclins B, C, D1 and D2), which would be downregulated or suppressed in advanced disease [14], [31], [35]. Similar to reactive BM, the higher proliferation of the more mature neutrophil lineage cells observed in MDS probably reflects an attempt to counteract neutropenia [8] by abnormally enhancing the proliferation capability of more differentiated cells [36]. In turn, the progressive collapse of cell proliferation in advanced/high-risk MDS cases could directly or indirectly be related to the accumulation of secondary genetic lesions and/or a progressively impaired BM stroma and cytokine production/response by the dysplastic hematopoietic BM precursors. In line with these observations, we have also recently found increased numbers of early CD34^+^/CyMPO^+^ neutrophil and CD34^+^/CD36^+^/CD123^lo^ erythroid precursors within the BM CD34^+^ HPC from early/low-risk MDS, while in advanced/high-risk MDS neutrophil and erythroid differentiation of CD34^+^ cells typically appeared to be blocked [37]. Of note, similar patterns of evolution were observed when analysis of cell proliferation was performed in paired diagnostic and follow-up samples from a subgroup of 11 MDS patients.

Altogether, these findings suggest that an increased proliferation of neutrophil and erythroid precursors, already detectable among CD34^+^ non-lymphoid (uncommitted plus myeloid) HPC in the early stages of the disease, could reflect an attempt of the BM to maintain peripheral counts of mature red cells and neutrophils, required for the subject to remain alive. Conversely, the impaired decreased proliferation of early myeloid precursors, particularly of the neutrophil and erythroid lineage, could represent an early event during progression of early/low-risk to advanced/high-risk MDS and a surrogate marker for a progressively increased risk of transformation to AL, further leading to the worsening of cytopenias observed in advanced disease. The acquired secondary genetic alterations and hypermethylation patterns observed among CD34^+^ and maturing BM cells from advanced MDS [32], [36] might further lead to abnormal expression of genes associated with basic cell functions (e.g.: Ankaryn 1 and Tropomodulin in the erythroid lineage) [38], consequently contributing to a gradual defective capacity for multilineage proliferation and differentiation of BM precursors [24], [37]. Overall, our findings point to a potentially altered response of advanced MDS patients to cell cycle and apoptotic mediators, specially among non-lymphoid (uncommitted plus myeloid) CD34^+^, NRBC and immature neutrophil precursors, whereas the more mature neutrophils might retain residual susceptibility to such proliferation stimuli. Noteworthy, MD/MPN patients showed a BM proliferation profile similar to that of early/low-risk cases, except for a slightly increased PI among monocytic precursors. Overexpression of cell-cycle proteins (e.g.: cyclin D1) specifically found among these patients could contribute to this unique profile [31]. Of note, our results could be viewed as apparently controversial since they show a progressively decreased cell proliferation of CD34^+^ cells in association with a higher accumulation of blast cells from low- to high-risk MDS and AML. However, they probably indicate that despite being highly proliferative, blast cells from low-risk MDS still retain their capacity to differentiate into more mature cells and therefore they do not accumulate at this stage. Conversely, high-risk MDS blast cells lose their ability to differentiate into more mature cells, such maturation blockade being associated with a numerical expansion of cells with increased survival and decreased apoptotic signalling, which most probably reflects both a decreased ability to maturate and also to proliferate, as both cell functions are intimately linked during hematopoiesis.

Based on all the above it could be expected that the impaired proliferation of distinct BM cell compartments in MDS could also be associated with individual prognostic factors, as well as with patient outcome. In detail, a significant association was specifically found between a low PI of non-lymphoid CD34^+^ precursors and NRBC and other adverse features of the disease (e.g.: higher number of cytopenias, anemia, thrombocytopenia, increased serum LDH, transfusion requirements and intermediate/poor cytogenetics) as well as with a worse disease outcome -shorter OS and progression to AL free survival (PFS)-. Interestingly, the impact of the degree of impairment of the proliferation of these two subsets of BM precursors in the outcome of MDS patients was retained when early/low-risk and advanced/high-risk MDS patients were separately considered. Even more, multivariate analysis of prognostic factors showed that a decreased PI of NRBC was the most powerful independent prognostic factor for both OS and acute leukemia PFS of MDS patients.

Altogether, these results suggest that assessment of the PI of NRBC, and potentially also of other compartments of BM precursors (e.g.: myeloid CD34^+^ HPC), could significantly contribute to a better management of MDS patients, potentially also during monitoring of the effects of new drugs and therapeutical strategies.

In summary, our results show the existence of altered proliferation profiles in the BM of MDS patients which are associated with unique but different patterns in early/low-risk *vs.* advanced/high-risk MDS. From the prognostic point of view, an abnormally low PI of non-lymphoid CD34^+^ cells and NRBC were both associated with a significantly longer OS and decreased risk of transformation to AL, even when early/low-risk and advanced/high-risk MDS patients were separately considered, the PI of NRBC emerging as an independent prognostic factor for both OS and PFS in MDS.
